# MVA-Vectored Pentameric Complex (PC) and gB Vaccines Improve Pregnancy Outcome after Guinea Pig CMV Challenge, but Only gB Vaccine Reduces Vertical Transmission

**DOI:** 10.3390/vaccines7040182

**Published:** 2019-11-14

**Authors:** Heidi Contreras, Felix Wussow, Claudia Fernández-Alarcón, Craig Bierle, Jenny Nguyen, Don J. Diamond, Mark R. Schleiss

**Affiliations:** 1 Department of Hematology & HCT, City of Hope, Duarte, CA 91010, USA; hcontreras@coh.org (H.C.); jenguyen@coh.org (J.N.); DDiamond@coh.org (D.J.D.); 2 Division of Pediatric Infectious Diseases and Immunology, Department of Pediatrics, University of Minnesota Medical School, Minneapolis, MN 55455, USA; ferna128@umn.edu (C.F.-A.); cjbierle@umn.edu (C.B.)

**Keywords:** congenital cytomegalovirus infection, cytomegalovirus vaccine, pentameric complex (PC), cytomegalovirus glycoproteins, guinea pig cytomegalovirus

## Abstract

(1) Background: A congenital cytomegalovirus (cCMV) vaccine is a major research priority, but the essential glycoprotein target(s) remain unclear. We compared CMV gB (gpgB), gH/gL (gp75/gL), and pentameric complex (gpPC, composed of gH/gL/GP129/GP131/GP133) vaccines in a guinea pig CMV (GPCMV) congenital infection model. (2) Methods: Modified vaccinia virus Ankara (MVA) vaccines expressing GPCMV glycoproteins were used to immunize GPCMV-seronegative, female Hartley guinea pigs (three-dose series, 3 × 10^7^ pfu/dose). After pregnancy was established, the dams underwent an early third-trimester challenge with salivary gland (SG)-adapted GPCMV. (3) Results: All vaccines elicited GPCMV-specific binding and neutralizing antibodies. Preconception immunization resulted in 19.5-, 4.9-, and 698-fold reductions in maternal DNAemia in MVA-gp75/gL, MVA-gpPC and MVA-gpgB groups, respectively, at day 14, post-SG challenge. Vaccination improved pups’ birth weight and reduced mortality and congenital CMV transmission. In controls, cCMV infection was observed in 100% of pups (mean viral load in all visceral organs, 2.4 × 10^4^ genomes/mg), versus 50% in the gB group (visceral viral load, 9.4 × 10^2^ genomes/mg; *p* < 0.05). No significant reductions in congenital transmission were noted in the MVA-gp75/gL and MVA-gpPC groups. (4) Conclusions: MVA-vectored gB, gH/gL, and PC vaccines were immunogenic, and protected against maternal DNAemia and pup mortality. These results support the inclusion of multiple glycoprotein complexes in a cCMV vaccine.

## 1. Introduction

Human cytomegalovirus (HCMV) is the most common congenital viral infection globally, and is a major cause of neurodevelopmental delays and long-term disability [1,2]. HCMV is also a leading cause of nongenetic sensorineural hearing loss worldwide [3,4]. Development of a vaccine against congenital CMV (cCMV) is a major public health priority, but, to date, there is no licensed vaccine available. A major challenge in vaccine development is the fact that the protective correlate(s) of immunity, essential for preventing infection of the developing fetus, remain unknown [5,6]. Moreover, it is not clear if a vaccine platform should be based on a live, attenuated strain of HCMV; disabled, replication-deficient HCMV genomic variants; or subunit vaccines delivering specific viral immunogens (either as adjuvanted purified proteins, or vectored through heterologous expression systems) that are believed to be important in protective immunity. Several HCMV vaccines based on these, and other, strategies are currently in various phases of preclinical and/or clinical trial evaluation [7]. 

Of the three HCMV subunit vaccines that have been evaluated in Phase II clinical trials [8], the HCMV envelope glycoprotein B (gB), administered with a squalene-based, oil-in-water emulsion adjuvant known as MF59, has received the most attention [9,10]. This vaccine is the only platform to date that has demonstrated efficacy in prevention of primary HCMV infection in women of potential child-bearing age [9,10]. When administered with MF59, the gB vaccine demonstrated 43–50% efficacy in phase II clinical trials in populations of seronegative postpartum [10,11] and adolescent women [12]. Surprisingly, recent evidence suggests that the correlate of protection elicited by the gB/MF59 vaccine was not a virus-neutralizing antibody, but a non-neutralizing function: antibody-dependent cellular phagocytosis (ADCP) [13,14]. These observations have stimulated interest in efforts aimed at further refining gB subunit vaccines. Strategies include the inclusion of other immunogenic HCMV antigens, in addition to gB; the exploration of more potent adjuvants; and the development of genetic and biochemical approaches that might optimize immune responses to the most immunogenic conformations and epitopes of the gB molecule [15,16]. 

Several additional HCMV glycoproteins and glycoprotein complexes have been proposed, as additions and/or alternatives to gB, for prophylactic subunit vaccination to protect against cCMV transmission. HCMV utilizes different glycoprotein complexes to infect different cell types: entry into fibroblasts involves glycoprotein homotrimers gB and gH/gL/gO [17,18,19], whereas entry into epithelial and endothelial cells requires gB, gH/gL/gO, and the pentameric complex (PC, gH/gL/UL128/130/UL131A) [20,21,22,23]. The discovery that high potency anti-PC antibodies neutralize HCMV infectivity [24,25] has rekindled interest in the potential clinical utility of high-titer anti-HCMV immune globulins. Recent studies have demonstrated that the majority of the virus-neutralizing activity in Cytogam^®^, (CSL Behring LLC, King of Prussia, PA, USA), a commercially prepared, pooled, high-titer human convalescent sera, targets the PC, and not gB [26]. These observations have, in turn, driven interest in devising expression strategies targeting the PC in subunit vaccine design. Strategies include the expression of the PC as a component of a disabled, infectious, single-cycle (DISC) HCMV vaccine [27]; the purification of recombinant PC protein from CHO cells [28]; and the expression of PC in a vectored vaccine, using recombinant modified vaccinia virus Ankara (MVA; [29]). PC-based vaccines are in several stages of development, but none have yet been tested in clinical trials. An initial report suggested an association between rapidly-developing high-titer, anti-PC antibodies and improved pregnancy outcomes [21]. Thus, the rapidity of induction of anti-PC antibody may be a critical requirement for such a vaccine. However, not all studies report a protective benefit of anti-PC antibodies against cCMV transmission. For example, in a recent report from Brazil, the titer of anti-PC and anti-gH/gL/gO antibodies did not correlate with protection against cCMV in a high-seroprevalence population of women of childbearing age [30].

Given the uncertainty about which glycoprotein complex(es) represent the ideal immunogen(s) for inclusion in a subunit cCMV vaccine, it would be desirable to compare the protective efficacy of gB- and PC-based vaccines in an animal model of cCMV transmission. This information would help inform and direct future clinical trials. Since CMVs are highly species-specific, HCMV vaccines cannot be evaluated for protection against cCMV in any animal model. However, both rhesus macaque CMV (RhCMV) and guinea pig CMV (GPCMV) provide useful models for vaccine study [31,32]. Moreover, both viruses encode homologs of the PC. In addition to gH and gL, Rh157.5, Rh157.4, and Rh157.6 encode proteins that are homologs of HCMV UL128, UL130, and UL131 [33]. As with HCMV, the RhCMV PC is essential for entry into epithelial cells [34,35]. In rhesus macaques vaccinated with a bacterial artificial chromosome (BAC)-derived MVA vector co-expressing all five RhCMV pentameric subunits, potent RhCMV-specific neutralizing antibody responses were elicited that were capable of blocking infection of epithelial cells and fibroblasts. Reduced RhCMV plasma viral loads were also observed following vaccination and subsequent RhCMV challenge [36]. Notably, GPCMV also encodes homologs of the HCMV PC ORFs [37,38,39,40], including gH, gL, and proteins designated GP129/131/133 (homologs of HCMV UL128/130/131a). Although the GPCMV PC elicits antibody responses in the context of GPCMV infection [39], no high-potency neutralizing monoclonal antibodies, specifically targeting conformational epitopes, have been described [41], and, to date, there has been no examination of a PC-based subunit vaccine to test for protection against congenital GPCMV transmission. Thus, this study was undertaken to compare the protective efficacy of a gB-based vaccine with gH/gL and PC-based vaccines, each generated using an MVA-vectored approach, in the context of the GPCMV congenital infection model.

## 2. Materials and Methods 

### 2.1. Guinea Pigs

Outbred Hartley guinea pigs were purchased from Elm Hill Laboratories (Chelmsford, MA, USA). Strain 2 guinea pigs (for propagation of salivary gland stocks) were maintained in the University of Minnesota (UMN) vivarium. All animals were confirmed to be GPCMV-seronegative by ELISA [42] and housed under conditions approved by the Institutional Animal Care and Use Committee (IACUC) at the UMN, Minneapolis (protocol 1607-33994A, initially approved in 2016, and renewed by the IACUC in 2019).

### 2.2. Cells and Virus

Salivary gland-passaged GPCMV virus stocks were prepared in strain-2 guinea pigs [42]. Baby Hamster Kidney (BHK) cells were maintained as previously described [42]. Cell culture for GPCMV was carried out in guinea pig lung fibroblast cells (GPL; ATCC CCL158) in F-12 medium, supplemented with 10% fetal calf serum (FCS, ThermoFisher Scientific, Waltham, MA, USA) 10,000 IU/l penicillin, 10 mg/l streptomycin (Gibco-BRL) and 0.75% NaHCO3 (Gibco-BRL). For neutralization assays, an eGFP-tagged virus with an intact PC was used, as previously described [42].

### 2.3. Generation of Vaccine Constructs and Western Blot Analyses

To create the vaccines, 2A sequences were used to facilitate expression of the PC subunits in the context of recombinant MVA [36,43,44]. To evaluate whether a multicistronic GPCMV PC construct could direct synthesis of the intact complex, a transient transfection assay was performed using a plasmid, pKTS 885, that directed synthesis of the GP75, GP115, GP129, GP131, and GP133-v5-His ORFs under the control of the HCMV major immediate early promoter (MIEP). Table 1 reviews the characteristics of the GPCMV homolog ORFs, relative to their HCMV counterparts. The pKTS 885 construct was engineered with 2A sequences inserted between each individual ORF, and the terminal ORF, GP133, was tagged with V5, to allow detection of the full complex. Following the transfection of plasmid into GPL cells, RIPA buffer was used to lyse cells, and anti-V5 was used to immunoprecipitate GP133-V5-His and interacting proteins, which were detected using an anti-2A antibody by western blot (described below).

**Figure 1:**
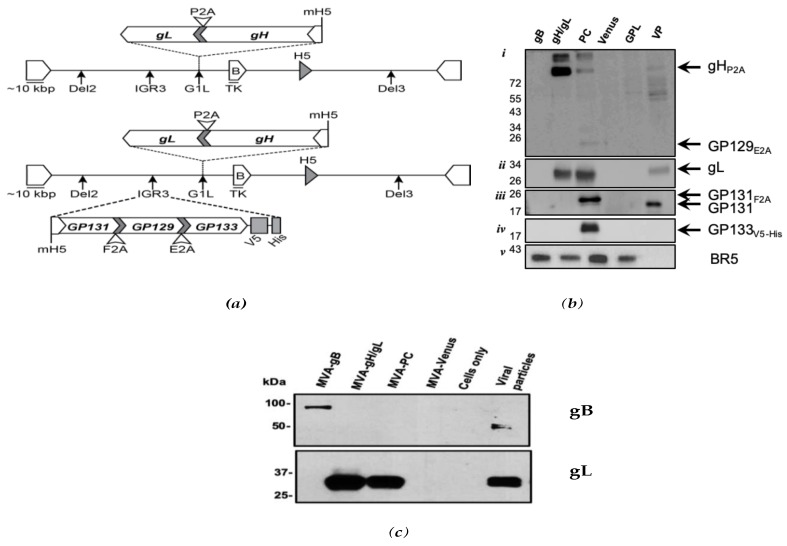
Modified vaccinia virus Ankara (MVA) expressing gpPC subunits. (**a**) Schematic representation of the gene distribution on MVA. Top: The genes for gH (GP75) and gL (GP115) are engineered into the G1L site on MVA, separated by a P2A cleavage peptide. Gene expression is under the control of the modified H5 (mH5) promoter. Bottom: Genes GP131, GP129, and GP133 were added into the IGR3 site of MVA harboring gH/gL, in the G1L site. Each subunit is separated by unique 2A sequences; GP133 has a tandem C-terminal V5-His tag. (**b**) Guinea pig fibroblast lung cells (GPL) cells were infected with MVAs that expressed gB, gH/gL, or PC ORFs. Arrows indicate the position of gH, GP129, gL, GP131, and GP133. All samples are GPL cell lysates infected with MVA-gpgB (gB), MVA-gp75/gL (gH/gL), MVA-gpPC (PC), MVA-Venus (Venus, negative control), mock-infected cells (GPL), or GPL cells infected with GPCMV viral particles (VP, positive control) from salivary gland (SG)-adapted cell supernatants. Panels *i–v*, western blots with individual antibodies, as described in text. BR5 antibody against MVA was used as an MVA infection control. (**c**) Western blots of lysate from MVA-inoculated BHK cells and of purified GPCMV viral particles (included as positive controls). The expression of gB in the MVA-gpgB vectored vaccine lysate (~1 μg/well) is noted as an uncleaved polypeptide, due to the absence of the furin cleavage site in the MVA-gpgB recombinant [42]; in contrast, gB moab 29–29 (which has a specificity in the COOH-terminal moiety of gB) identifies a ~58 kDa subunit in GPCMV particles.

Next, modified vaccinia virus Ankara (MVA) vectors, expressing gB, gH/gL, or the GPCMV PC subunits, were engineered (Figure 1a). Details regarding the generation of the gB MVA (MVA-gpgB) construct have been previously published [42]. The gH/gL (gp75/gL) and PC (gpPC) constructs were generated using our previously generated MVA BAC vector, termed MVABAC-TK, and en passant BAC mutagenesis [43,44]. MVA-gp75/gL and gpPC both contain a 2A-linked gH/gL subunit, inserted into the G1L site [45]. MVA-gpPC also contains 2A-linked GP129/GP131/GP133 subunits, inserted into the IGR3 site [46]. The 2A-linked gH/gL and GP129/GP131/GP133 subunits, inserted into MVA, were derived from pKTS 855 (Figure 1). Expression of all GPCMV transgenes was driven by the modified vaccinia H5 promoter (mH5), which facilitates robust gene expression during early and late phases of the vaccinia virus replication cycle [47,48]. GP133 was C-terminally tagged with V5. Mutant MVA was plaque-purified and grown to high titer to generate vaccine stocks. 

To confirm expression of GPCMV antigens, western blot assays were performed using lysates from GPL cells infected with recombinant MVAs (Figure 1b), as previously described [42]. Western blot of MVA-infected and control lysates were performed using the following antibodies: a mouse monoclonal anti-V5 antibody (Sigma–Aldrich, St. Louis, MO, USA) for detection of GP133; mouse monoclonal anti-2A clone 3H4 (Millipore, Temecula, CA, USA) for detection of gp75 and GP129; affinity purified polyclonal antibody, #YZ6728, from a rabbit immunized with GP115 peptide #230-250 (YenZym; #YZ6728; peptide NH_2_-CVRRLILYQASLSGPHRDAPIHNYLNRDLS-COOH) for detection of GPCMV gL; and an affinity purified rabbit polyclonal antibody, against GP131, US818 [39]. For MVA infection control, vaccinia virus BR5 was detected using moab 19C2 [49]. An anti-V5 tag antibody-ChIP Grade (ab9116) Abcam antibody was also used for GP133/V5-His detection for some experiments (data not shown). 

We also qualitatively assessed whether the MVA vaccines produced similar amounts of gB, gH/gL or PC by western blot, when expressed in BHK cells. BHK cells were inoculated with either MVA-gpgB, MVA-gp75/gL, or MVA-gpPC recombinant vaccine, and lysates harvested as previously described [42]. SDS-PAGE and western blots were analyzed using either the anti-gL antibody, as described above, or a moab against GPCMV gB [42]. These comparisons suggested that the total amount of recombinant immunogen was qualitatively similar in the MVA-vectored vaccines (Figure 1c).

### 2.4. Mass Spectrometry Analyses

For sample preparation for mass spectrometry analyses, immunoprecipitation (IP) was performed as previously described [29]. Briefly, BHK cells were infected with MVA recombinants at ~90% confluence at a MOI of 0.1, then harvested after 16–20 h of incubation. The following steps were performed at 4 °C or on ice. Cells were lysed in 10 mL PBS, 10% Glycerol, 0.1% (*w*/*v*) TritonX-100, 0.1 mM PMSF and Complete Mini protease inhibitor cocktail tablets (Roche) buffer. Lysates were cleared by centrifugation at 10,000 × g and incubated two times for 4 h, with recombinant Protein G Agarose beads (Invitrogen, Carlsbad, CA, USA), to remove nonspecific binding, at 4 °C, on an orbital shaker. IP was performed by the incubation of pre-cleared lysates for 2 h to overnight, with recombinant Protein G Agarose beads, coupled to mouse anti-V5 mAb or anti-2A polyclonal antibodies. As a negative control, samples were incubated with beads coupled to a mouse IgG1κ control Ab (Biolegend, San Diego, CA, USA). The beads were washed six times with PBS and boiled in SDS sample buffer. Gel slices corresponding to estimated subunit molecular weight were in-gel trypsin digested and processed for mass spectrometry analysis. Immunoprecipitated proteins were detected by immunoblot, as described above. 

All MS/MS samples were analyzed using Sequest (Thermo Fisher Scientific, San Jose, CA, USA; version 2.1.0.81). Sequest was set up to search against a combined protein FASTA database with sequence entries from the NCBI Reference Sequence for *Mesocricetus auratus* [50], *Caviid betaherpesvirus 2* (GPCMV) [51], and common laboratory contaminants [52]. Sequest was searched with a fragment ion mass tolerance of 0.60 Da and a parent ion tolerance of 5.0 PPM. Carbamidomethyl of cysteine was specified in Sequest as a fixed modification. Oxidation of methionine and acetyl groups on the N-terminus were specified in Sequest as variable modifications.

Criteria for protein identification were performed using Scaffold (version Scaffold_4.8.4, Proteome Software Inc., Portland, OR, USA), and were used to validate MS/MS based peptide and protein identifications. Peptide identifications were accepted if they could be established at greater than 94.0% probability by the Scaffold Local FDR algorithm. Protein identifications were accepted if they could be established at greater than 99.0% probability, to achieve an FDR less than 1.0%, and contained at least four identified peptides. The Protein Prophet algorithm assigned protein probabilities [53]. Proteins that contained similar peptides, and could not be differentiated based on MS/MS analysis alone, were grouped to satisfy the principles of parsimony, and, thus, peptides were assumed to correspond to *Caviid betaherpesvirus* 2.

### 2.5. Immunization Schedule and Immune Assays

Guinea pigs were immunized with MVA-gpgB, MVA-gpgH/gL (gp75/gL), MVA-gpPC, or MVA-Venus. MVA-Venus, which expresses a yellow fluorescent protein, was used as the negative control. A total of eight animals/group were subcutaneously administered a three-dose series of vaccines (3 × 10^7^ pfu/dose) at a monthly interval, diluted as needed to a total volume of ~0.5 mL for each injection. Serum was collected one month after each immunization for serological analyses.

ELISA assays were performed as previously described [42]. ELISA titers were defined as the reciprocal of the highest dilution that produced an absorbance of at least 0.10, and twice the absorbance of a negative-control antigen, prepared from uninfected guinea pig lung (GPL) cells (ATCC CCL158). Titers of <40 were assigned a value of 20 for statistical comparison. The GFP-tagged recombinant GPCMV (vJZ848) virus was used for neutralization assays, using published protocols [42]. vJZ848 contains an intact, wild-type PC sequence [42]. Rabbit sera was used as a source of exogenous complement. Neutralizing titers were determined in assays using GPL cells. Neutralizing titers were defined as the dilution resulting in the reduction of ≥50% of the total number of GFP-positive foci. Western blots were performed using purified GPCMV virus particles as the target antigen, as described elsewhere [39], with monospecific rabbit anti-peptide antibodies targeting individual constituents of the GPCMV PC complex used as controls. 

Guinea breeding was commenced within 14 days after the third vaccination. Pregnancies were monitored by palpation, and dams were challenged in the early third trimester with SG-adapted GPCMV, at a dose of 1 × 10^5^ PFU, by subcutaneous route [42]. Pregnancy outcomes (maternal viremia, birth weights, live/dead pups, and congenital infection rates) were then monitored. All live-born pups were sacrificed within 72 h of delivery for organ harvest and PCR analysis, with comparisons made to visceral organs of still-born pups, harvested at the time of delivery.

### 2.6. Real-Time qPCR Analysis

Maternal blood was obtained on days 7 and 14 post-challenge, with SG-adapted virus, and analyzed for viral load by qPCR, as described previously [42]. Briefly, DNA was extracted from either 100 μl citrated maternal blood, or from pup tissues, using 0.05 g of homogenized frozen samples of liver, lung or spleen (QIAamp 96 DNA QIAcube HT Kit, Qiagen, Hilden, Germany). Amplification primers GP83TM_F1 (5’-CGTCCTCCTGTCGGTCAAAC-3’) and GP83TM_R1 (5’-CTCCGCCTTGAACACCTGAA-3’) were used at a final concentration of 0.4 µM, while the GP83 hydrolysis probe (FAM-CGCCTGCATGACTCACGTCGA-BHQ1) was used at 0.1 µM. PCR was performed as previously described [42], and data were analyzed with the LightCycler Data Analysis Software (version 1.5; Roche), using standard curves generated from known copy numbers of a modified plasmid pCR 2.1 containing GP83 sequences. DNAemia was expressed as the total number of genome copies per mL of blood (limit of detection ~200 copies/mL). For the purpose of statistical comparison, a level of 100 copies/mL was assigned to negative samples. Tissue viral loads were expressed as genome copies per mg of tissue. The limit of detection was approximately two genome copies/mg tissue. For the purpose of statistical comparisons, a value of one copy/mg was assigned to negative samples.

### 2.7. Statistical Analyses

GraphPad Prism software (version 8.0, San Diego, CA, USA) was used for statistical analyses. Pup mortality and transmission data were compared using Fisher’s exact test with one-sided comparisons. Antibody titers and visceral viral loads in pups were compared using non-parametric comparisons (Mann–Whitney and Kruskall–Wallis, with Dunn’s multiple comparisons). Parametric data (pup birth weights) were compared using two-way analysis of variance (ANOVA), with values adjusted by Bonferroni’s multiple-comparison method.

## 3. Results 

### 3.1. Expression of GPCMV PC Subunits in MVA

Two MVA constructs were engineered to express GPCMV PC constituents. Subunits were inserted into two MVA insertion sites: (1) gH and gL (gp75/gL) in G1L; and (2) GP131, GP129, and GP133V5-His in IGR3 (Figure 1a). BAC clones of MVA were used to express self-processing subunits of PC subunits, facilitated by unique 2A sequences (Table 2). The “ribosomal skipping mechanism,” mediated by 2A peptides [43], was exploited to generate MVA vectors expressing all five GPCMV PC subunits (MVA-gpPC), or only GPCMV gH/gL (MVA-gp75/gL) (Figure 1a). Anti-2A antibody was raised against the epitope GDVESNPGP of foot-and-mouth picornavirus F2A 2A and is cross reactive with other 2A peptides. Thus, anti-2A can recognize the C-terminal peptide on GP75, GP131, and GP129 (Table 2). Glycoproteins gH/gL (MVA-gp75/gL) were expressed under the control of a modified H5 (mH5) promoter while GP131, GP129, and GP133V5-His (MVA-gpPC) were controlled by their own mH5 promoter. Two insertion sites, G1L and IGR3, were chosen gp75/gL and GP129/GP131/GP133V5-His expression, respectively, based on observations that these sites in MVA stably harbor genes for prolonged viral passaging, without inducing mutations. 

Recombinant viruses MVA-gpPC and MVA-gp75/gL were used to infect GPL cells to confirm expression of GPCMV antigens. Protein lysates were probed with monoclonal anti-gB antibody 29.29 for gB expression (not shown), affinity purified rabbit antibody #YZ6728 (YenZym, Rabbits immunized with GP115 #230–250) for gL expression, and anti-V5 (for detection of C-terminal V5 on GP133). Because we were unable to isolate mono-specific antibodies against all individual subunits, we utilized the 2A peptides located on the C-terminus of gH, GP131, and GP129, to detect subunits that had no corresponding antibody reagent (Figure 1b). In Figure 1b, individual antibodies detected MVA-infected GPL cells, expressing all five gpPC subunits. The bottom three panels of Figure 1b show expression of gHP2A and GP129E2A, GP131F2A, and GP133V5-His. Individual expression profiles were as follows: (*i*) the top panel demonstrates the expression of gH and GP129 at ~84 kDa and ~22.8 kDa, respectively, due to 2A peptides on the C-terminus of either protein; (*ii*) the second panel demonstrates the expression of gL in the gH/gL constructs and in the positive control lane of GPCMV viral particles [VP]; (*iii*) the third panel shows that GP131 expressed from MVA appears to have a higher molecular weight than that observed in the virus particles, possibly due to posttranslational modifications; (*iv*) the fourth panel shows confirmation of the expression of the GP133 protein, demonstrated by probing for the C-terminal V5 tag; (*v*) the fifth panel contains the BR5 immunoblot, which shows equivalent loading of wells for MVA-infected cells. We also qualitatively assessed whether the MVA vectored vaccines produced similar amounts of recombinant GPCMV proteins, by western blot analysis, BHK cells. This also addressed whether the MVA vaccines produced similar amounts of gB, gH/gL or PC, in order to gauge whether differences in vaccine immunogenicity were due to differences in protein expression in the respective MVA constructs. As shown in Figure 1c, these data suggested that there were similar levels of expression of the constituents of PC proteins (as assessed by gL antibody), and of gB, driven by the recombinant MVA vaccines.

**Figure 2:**
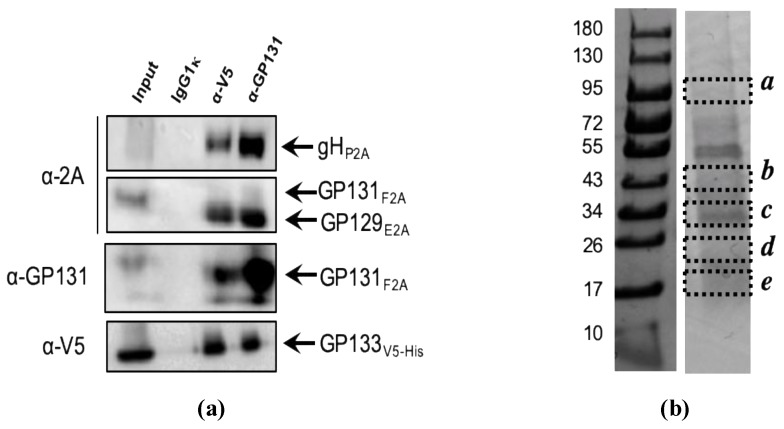
Co-immunoprecipitation of gpPC, expressed in BHK-infected with MVA expressing recombinant gpPC. (**a**) Co-immunoprecipitation of MVA-expressing gpPC subunits. Protein was enriched from whole cell lysate (Input) by precipitation via the following antibodies: IgG1κ (negative control), α-V5 (monoclonal), and α-GP131 (rabbit polyclonal). For the detection of individual subunits, western blot analysis was performed, with respective antibodies. Values on the left denote molecular weight in kDa. (**b**) Proteins were separated on a 4–20% SDS-PAGE gradient gel (Bio-Rad, Hercules, CA, USA). The gel was Coomassie-stained with Code Blue (Thermo Fisher Scientific). Dashed boxes indicate excised gel slices, each with unique letter identifiers. In-gel trypsin digestion was performed. Peptides were analyzed as described in Materials and Methods.

To further confirm the presence of a gpPC complex formation, recombinant MVAs were used to infect BHK cells for co-immunoprecipitation (co-IP) studies. Co-IPs were performed with either α-V5 monoclonal or α-GP131 polyclonal antibodies (Figure 2a); co-IP with IgG1κ was also performed as a negative control. We were able to identify 4/5 subunits of gpPC from co-IP studies via western blot. To identify the presence of GP115, mass spectrometry analysis was performed after co-IP of the complex. Co-IP samples were resolved and visualized on a Coomassie-stained 12% SDS-PAGE. Gel slices corresponding to the estimated molecular weights of the PC subunits were cut and analyzed (Figure 2b; Table 3). Of the five bands, gH, gL, and GP133V5-His were identified (Table 3). Although GP131 and GP129 were not identified by mass spectrometry analysis, we were able to observe them via western blot analysis. Furthermore, it has been suggested that GP133 will not complex with the gH/gL heterodimer in the absence of the GP131 and GP129, due, in part, to the requirement of the C-terminus of GP131 and GP133 [54], although subcomplex formation cannot be ruled out [38]. Therefore, these results indirectly suggest that there is formation of a gpPC complex when these ORFs are expressed in the MVA vectored vaccine construct. 

### 3.2. gB Vaccine Elicits Higher ELISA and Neutralization Responses than gH/gL or PC

**Figure 3:**
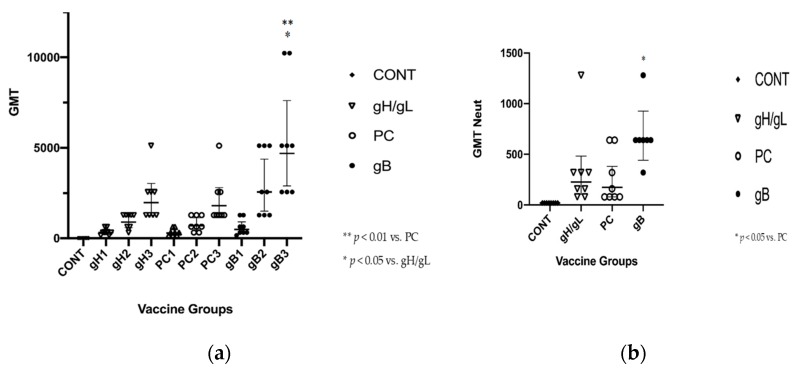
ELISA and neutralizing antibody responses after MVA vectored glycoprotein vaccination. GPCMV-seronegative Harley guinea pigs (*n* = 8/group) were immunized with a three-dose series of MVA vaccines. Groups consisted of MVA-Venus (vector only control); MVA-gp75/gL (gH/gL) vaccine; MVA-gpPC vaccine; and MVA-gpgB vaccine. Following completion of the vaccine series, both (**a**) total ELISA anti-GPCMV antibody and (**b**) GPCMV virus-neutralizing antibody titers (methods described in Section 2.5) were determined. Geometric mean titer (GMT) is shown for both ELISA antibody and neutralization (GMT-Neut) titers. Data are plotted as individual data points; statistical comparisons were performed by nonparametric analysis, and P values are as indicated.

In order to assess the comparative immunogenicity of gB, gH/gL, and PC vaccines, responses were measured approximately four weeks after each dose of glycoprotein subunit (or control) vaccine (Figure 3a). All animals (8/8 in the gH/gL and PC groups, and 7/7 in the gB group) had low-level ELISA responses after the first dose of the vaccine, with titers rising after subsequent immunizations, through to the completion of the vaccine series. After a second dose of vaccine, statistically significant differences in ELISA titer were noted, upon comparison of the gB vaccine with both the gH/gL vaccine and the PC vaccine. Geometric mean titer (GMT) after two doses was 905.1 (95% CI, 584.1–1403) in the gH/gL group; 697.9 (95% CI, 430.1–1132) in the PC group; and 2560 (95% CI, 1497–4378) in the gB group (*p* < 0.001 vs. gH/gL and PC). Upon completion of the three-dose series, GMT in animals immunized with gH/gL was 1974 (95% CI, 1283–3038); for PC-vaccinated animals, GMT was 1810 (95% CI, 1168–2805); for gB, GMT was 4695 (95% CI, 2895–7615; *p* < 0.01 v. PC dose 3, *p* < 0.05 v. gH/gL dose 3). 

Comparison of neutralization titers (measured after three doses of vaccine) across the groups demonstrated similar differences in immunogenicity, with increased neutralization responses noted in the gB vaccine group. All animals (8/8 in the gH/gL and PC groups, and 7/7 in the gB group) had neutralization responses after completion of the vaccine series, although titers varied from animal to animal (Figure 3b). The GMT neutralization titer in animals immunized with the gH/gL construct was 226.3 (95% CI, 10.89–669.1); for PC-vaccinated animals, GMT neutralization titer was 174.5 (95% CI, 52.31–467.7); and for gB, the titer was 640 (95% CI, 419.4–952). Mann–Whitney analysis indicated that there were statistically significant differences in the neutralization titer for the gB-immunized group, when compared to the PC vaccinated animals (*p* < 0.05). No neutralizing responses were noted in the control-immunized animals.

### 3.3. Impact of Preconception Vaccine on Maternal Viremia After GPCMV Challenge

In order to assess the protective effect of preconception vaccination on adverse pregnancy outcomes following an early third trimester challenge with virulent SG-adapted GPCMV, immunized dams were challenged, and outcomes were compared [55]. Briefly, in this model, animals are mated, and pregnancies monitored by palpation, using previously published methods [42]. Based on experience gleaned from past mating studies and palpation, we estimated the onset of the third trimester (a typical guinea pig pregnancy is approximately 65 days in duration [32]), and targeted this time point for subcutaneous viral challenge with SG-adapted virus. Real-time PCR monitoring of viral load was performed at days 7 and 14 post-viral challenge, as described in Section 2.6, and pregnancy outcomes were analyzed. 

**Figure 4:**
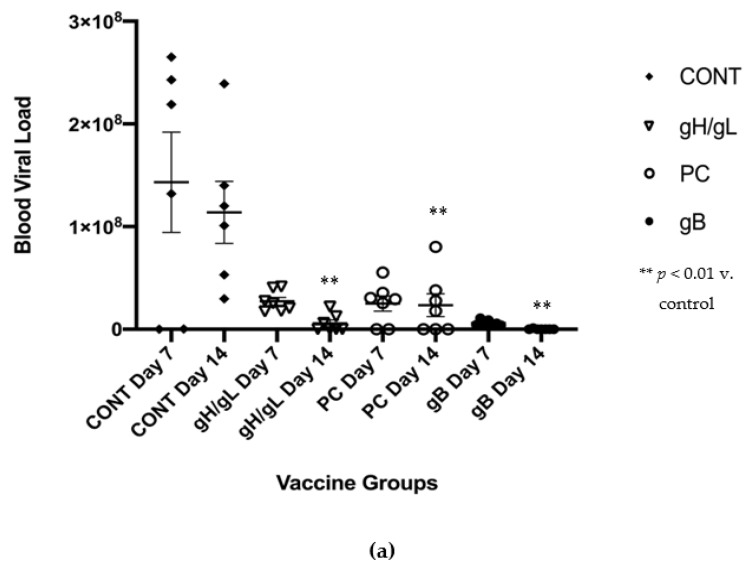

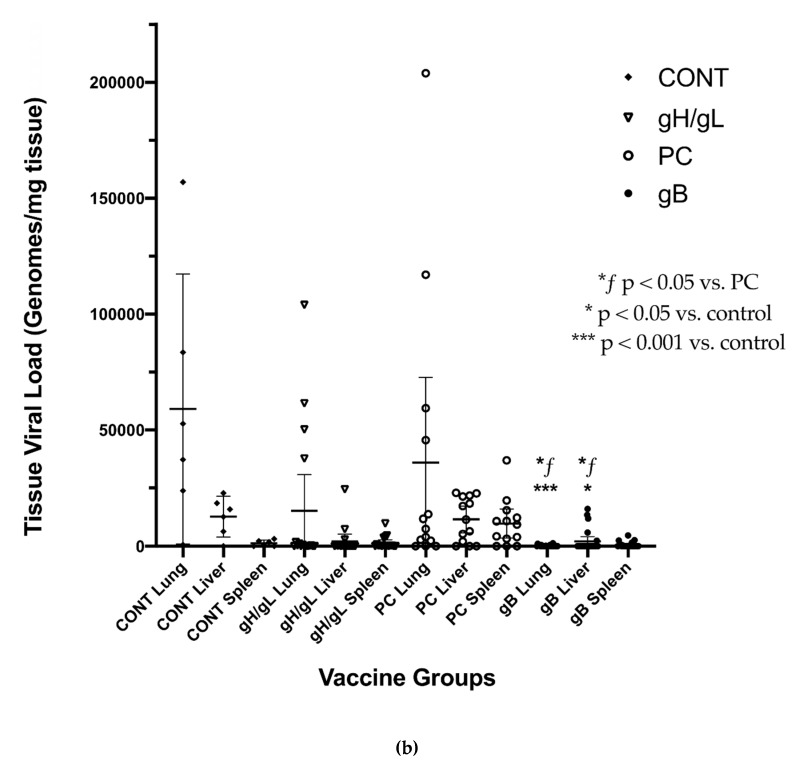
Impact of preconception vaccination on maternal viremia and congenital GPCMV transmission. (**a**) Comparison of maternal DNAemia (genome copies per ml) following challenge with virulent SG-GPCMV. Whole blood was obtained at 7- and 14-days post-challenge and evaluated by real-time PCR as described in text. Significant reductions in maternal DNAemia were noted at day 14, in dams immunized with all three vaccines (*p* ≤ 0.01), as assessed by nonparametric (Mann–Whitney) analyses. (**b**) The capability of MVA vaccines to reduce the pup viral load following the maternal challenge was analyzed by examining visceral organs (lung, liver, spleen) from pups, and quantifying GPCMV copy number (genome copies/mg tissue extracted) by real-time PCR. Data are represented as mean ± SEM. Significant differences, as assessed by Kruskall–Wallis and multiple comparisons, are noted: ***, *p* < 0.001 v. control; *, *p* < 0.05 versus control; * *f*, *p* < 0.05 v. PC.

For each of the gp75/gL, gpgB and gpPC groups, seven out of eight animals became pregnant. In the control (Venus) group, six animals became pregnant. Maternal viremia, pup mortality, and pup birth weight were compared across all groups, and vertical transmission rates were compared for those pups born to dams >7 days post-SG virus challenge (described below). Preconception immunization resulted in 19.5-, 4.9-, and 698-fold reduction in maternal DNAemia at day 14, post-challenge (Figure 4a) in the gp75/gL, gpPC, and gpgB groups, respectively, compared to MVA-Venus controls. For the control dams, the mean viral blood viral loads were 1.4 ± 0.5 × 10^8^ and 1.1 ± 0.3 × 10^8^ genomes/mL ( ± SEM) at days 7 and 14, respectively. In gp75/gL-vaccinated dams, viral load was reduced to 2.7 ± 0.4 × 10^7^ (*p* = NS vs. control) and 5.8 ± 3.3 × 10^6^ (*p* = 0.01 vs. control) genomes/ml at days 7 and 14; in gpPC-vaccinated animals, viral load was 2.5 ± 0.7 × 10^7^ and 2.3 ± 1.1 × 10^7^ genomes/ml at days 7 and 14 (day 14, *p* < 0.01 vs. control by Mann–Whitney comparison to control), respectively; and for gpgB-vaccinated dams, viral load was 6.1 ± 1.0 × 10^6^ (*p* = NS vs. control) and 1.6 ± 1.6 × 10^5^ genomes/ml (*p* < 0.001 vs. controls) at days 7 and 14. Thus, all vaccine strategies resulted, in varying degrees, in a reduction in the magnitude of maternal DNAemia.

### 3.4. Impact of Preconception Vaccine on Pup Birth Weight, Mortality, and Congenital Infection 

**Figure 5:**
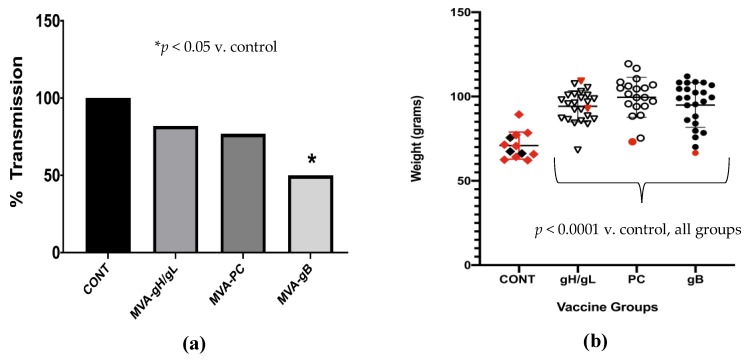
Pup outcomes following MVA vaccination. (**a**) The ability of the MVA vaccines to protect against congenital GPCMV transmission was analyzed by examining visceral organs (lung, liver, spleen) from pups, quantifying GPCMV copy number (genomes/mg tissue), and determining the congenital transmission rate, as described in text. Vertical transmission was observed in 100% of evaluable pups born to control dams; 82% of evaluable pups in the gp75/gL group; 77% of evaluable pups from the gpPC group; and 50% of evaluable pups in the gpgB group (* *p* < 0.05, Fisher’s exact test). (**b**) Weights of pups born to vaccinated guinea pigs, after challenge with GPCMV. Ordinate (y-axis) demonstrates pup weights (in grams) at time of delivery. Dead (stillborn) pups are symbolized by the red shapes; live-born pups are indicated by black shapes. For each of the three vaccine groups (MVA-gp75/gL [gH/gL], MVA-gpPC, and MVA-gpgB), there was a statistically significant improvement in pup birth weights (all *p* < 0.0001) compared to MVA-Venus immunized controls.

To assess the impact of vaccination on vertical GPCMV transmission, pups were dissected and DNA was extracted from liver, spleen, and lung homogenates. Pups were considered evaluable for congenital (transplacental) transmission if they were delivered > 7 days after the maternal SG virus challenge. If any of the three tissues demonstrated a positive signal by real-time PCR, that pup was designated as having congenital GPCMV transmission. Vertical transmission (Figure 5a) was observed in 100% of evaluable pups born to control dams (6/6 pups, three independent litters); 82% of evaluable pups in the gp75/gL group (14/17 pups from three independent litters); 77% of evaluable pups from the gpPC group (10/13 from four independent litters); and 50% of evaluable pups (12/24 from six independent litters) in the gpgB group (*p* < 0.05, Fisher’s exact test). 

The mean viral loads for all positive visceral organs (summarized in Figure 4b) were determined. In control pups (17/18 total visceral organs positive by PCR) the mean viral load (genomes/mg tissue ± SEM) for lung was 5.9 ± 2.3 × 10^4^; for liver, 1.3 ± 0.3 × 10^4^; and for spleen, 1.3 ± 0.3 × 10^3^. For the gp75/gL group, the mean viral load for lung was 1.5 ± 0.7 × 10^4^; for liver, 2.1 ± 1.5 × 10^3^; and for spleen, 1.4 ± 0.6 × 10^3^. In the gpPC pups, the mean viral load for lung was 3.6 ± 1.7 × 10^4^; for liver, 1.1 ± 0.3 × 10^4^; and for spleen, 9.7 ± 2.8 × 10^3^. The mean viral load in the gpgB group for lung was 2.1 ± 0.7 × 10^2^; for liver, 2.1 ± 1.0 × 10^3^; and for spleen, 5.4 ± 2.3 × 10^2^. The viral loads for each individual group of organs from each group are also demonstrated graphically, in Figure 4b. By Kruskal–Wallis analysis with Dunn’s multiple comparisons, the organ-specific reductions in pup viral load were significant when gpgB pup lung and control lung were compared (*p* < 0.001); when gpgB pup liver and control liver were compared (*p* < 0.05); and when gpgB pup lung and liver viral loads were each separately compared with the gpPC lung and liver tissue, respectively (*p* < 0.05). When all visceral organ viral load values were combined for data analysis for each vaccine group, only the gpgB vaccinated pups had a statistically significant difference in the overall magnitude of visceral viral load, compared to control (MVA-Venus) pups (2.4 × 10^4^ genomes/mg in all controls, versus 9.4 × 10^2^ genomes/mg in gpgB vaccine group pups; *p* < 0.05, data not shown). Therefore, we conclude that, in the guinea pig model, the gpgB MVA-vectored vaccine was associated with a statistically significant reduction in both the rate of congenital GPCMV transmission, and the magnitude of viral load in pup tissue.

Pup birth weights (± SD) of both the vaccine and control groups were compared (Figure 5b). Pups born to the control group (MVA-Venus) pups had a mean birth weight of 70.9 ± 8 g, compared to 94.2 ± 9.2, 99.5 ± 11.8, and 95.0 ± 13.2, in gp75/gL (gH/gL), PC and gB groups (for all vaccine groups, *p* < 0.001 versus control). Thus, the maternal preconception vaccination with all three MVA-vectored vaccines resulted in improved pup growth in utero following SG virus challenge. 

When pup mortality was compared in all immunized and control groups, all MVA-vectored glycoprotein vaccines conferred a protective effect (Table 4). Pup mortality following the maternal GPCMV challenge was 9/12 (75%) in pups born to controls, compared to 1/24 (4%), 2/24 (10%), and 1/20 (5%) in the gB, gH/gL, and PC groups, respectively (all *p* < 0.001 vs. control). The duration of pregnancy following the SG-adapted virus challenge was not significantly different among groups.

## 4. Discussion

Against the backdrop of the urgent need to generate a vaccine capable of preventing the disabilities caused by congenital HCMV infection, there is considerable interest in developing a vaccine platform that targets the HCMV PC (gH/gL/UL128/130/131A). This interest is driven by the discovery that the PC is essential for viral entry into epithelial and endothelial cells [17,18,19,20], and the observation that antibodies to PC are capable of potently neutralizing HCMV, both in the context of natural infection and following subunit immunization of mice using HCMV PC vaccine constructs [21,22,23,24,25,26]. Accordingly, multiple investigators and vaccine manufacturers are pursuing strategies that focus on targeting the PC as a key immunogen [27,28,29]. Since the GPCMV recapitulates much of the biology of HCMV, including the propensity of the virus for trans-placental transmission to the developing fetus [32], we undertook these studies in order to compare the efficacy of GPCMV, PC and gB-based subunit vaccines against congenital infection and disease.

Like HCMV, the GPCMV encodes a PC, consisting of proteins gH, gL, and UL129/131/133 [37,38,39,40]. We elected to express the GPCMV PC as an MVA-vectored vaccine. This approach has been successfully employed to generate both RhCMV and HCMV PC-based vaccines [29,36], both of which have been shown to be immunogenic and capable of directing the synthesis of a functional pentamer. Generation of the multi-subunit vaccine was facilitated by the use of 2A peptides [44]. This approach also had the advantage of generating a PC-expressing construct that could be directly compared to other MVA vaccines (hence the use of the same expression platform), based on additional key immunogens of interest in CMV vaccine design, such as gB. Previous work with an MVA-vectored gB vaccine in the GPCMV model demonstrated a vaccine-mediated reduction in pup mortality and reduced congenital GPCMV transmission [42]. Since the GPCMV PC has been demonstrated to elicit antibody responses in the context of GPCMV infection [39], this experiment provided an opportunity to compare the relative protective effect provided by the gB and PC vaccinations, in this relevant preclinical model.

Interestingly, the comparative analysis demonstrated that the gB subunit vaccine was superior in stimulating correlates of protective immunity by varying degrees, compared to either the PC vaccine or the gH/gL vaccine (engineered to express only the gH/gL proteins, and not the GPCMV PC). The MVA-gpgB vaccine resulted in a statistically significant reduction, compared to controls, in maternal DNAemia at day 14, following an early third trimester challenge of pregnant dams with virulent SG-adapted GPCMV. The gB vaccine was also significantly associated with reduced congenital transmission, and lower visceral organ viral loads in pups (Figure 4b). However, although not significantly associated with reduced congenital GPCMV transmission, both the MVA-gp75/gL and MVA-gpPC vaccines were also associated with improved pup survival (Table 4) and improved pup birth weight (Figure 5). These observations may be relevant to HCMV vaccines. Although the often-stated metric of success for the licensure of a vaccine against congenital HCMV infection is prevention of congenital infection [56], a vaccine that reduces disease in the newborn infant, even if transmission occurs, could be valuable. Although controversial [57], there are lines of evidence that suggest that congenital infection occurring in the context of recurrent maternal infection (re-infection in the face of pre-existing maternal immunity) may be less likely to produce sequelae in infants [58] than transmission occurring in the context of primary maternal infection during pregnancy. 

The key effector of protection in this study may have been the antibody response, in particular the neutralizing antibody response to vaccination. We observed that the MVA-gpgB vaccine elicited both an enhanced ELISA response, as well as a significantly higher neutralizing response, compared to the MVA-gp75/gL and MVA-gpPC vaccines. We confirmed that the eGFP-tagged reporter virus used for the neutralization studies, vJZ848, encoded wild-type sequence in the GPCMV PC locus (data not shown). Accordingly, any differences in neutralizing titers in GPL cell lines was not due to a failure of the reporter virus to express the PC proteins. 

One potential shortcoming of this study is that the GPCMV PC may not completely represent a bona fide homolog of the HCMV PC, in terms of pathogenesis and immune response. The HCMV PC plays a key role in viral entrance into epithelial and endothelial cells, but is dispensable for its entry into fibroblasts. The GPCMV PC, in contrast, does not appear to confer the same cell-type specific tropism-directing functions for epithelial and endothelial cells as the HCMV PC [38]. Indeed, the GPCMV PC appears to play a critical role in entry into many, diverse cell types, since a GP129-133 deletion mutant demonstrates defects in both endothelial cell and fibroblast cell entry [38]. The GPCMV PC, in particular the GP129, also plays a role in macrophage-mediated dissemination of virus in vivo [59], possibly mediated through a putative CC chemokine function [54]. The GPCMV PC is also required for pathogenesis in experimentally challenged, non-pregnant animals [60]. Future work will be required to more fully characterize whether vaccination against the GPCMV PC exerts a beneficial effect by eliciting virus-neutralizing activity, or perhaps instead by inducing antibody responses that target an immunomodulatory or chemokine-like activity, conferred by the GP129 protein. It is also notable that, while there is a correlation between neutralization titres and protection in this study, this may not completely represent the mechanistic correlate of protection, since non-neutralizing functions, such as ADCP, have recently been described that appear to mediate the modest protection conferred by gB/MF59 in HCMV vaccine studies [13,14,15]. Such non-neutralizing effectors of protective immunity would be fruitful areas for future evaluation of the GPCMV congenital infection model. 

Recently, a disabled, single-cycle (DISC) vaccine against GPCMV was described [61], attenuated by virtue of a mutation introduced into the *GP85* gene. This vaccine elicited a broad repertoire of immune responses, including both humoral and cellular responses (in particular, CD4+ T cell responses to the GPCMV homolog of HCMV pp65). The vaccine provided excellent protection against congenital GPCMV transmission. This DISC vaccine had an intact GPCMV PC sequence, but the dependence of anti-PC responses on the magnitude of vaccine-mediated protection was difficult to gauge, since the PC responses occurred in the context of an additional, broad repertoire of other antibody and cellular responses to many viral antigens. Furthermore, clear evidence that would suggest the production of potent neutralizing antibodies against the GPCMV PC (similar to the previously isolated human antibodies that predominantly target conformational epitopes of the UL128/130/131A subunits of the HCMV PC) was absent. Even more importantly, the protective studies were compared to historical controls that were based on responses engendered against a PC-negative “first generation” DISC vaccine [62] using a different dose regimen. The authors noted that, although both two- and three-dose regimens were examined for immunogenicity with the DISC vaccine, protection studies for the first-generation vaccine were completed using only those dams immunized with the suboptimal, two-dose vaccine regimen [62]. These were nonetheless directly compared to pregnancy outcome results using the second-generation DISC vaccine which were obtained using an optimized three-dose regimen [61]. Thus, the two studies are not strictly comparable. A contemporaneous, side-by-side, three-dose comparison of PC-intact- and PC-deficient DISC vaccines would be required to definitively assess the importance of the PC for protection in the GPCMV model. 

In summary, the data from this study support the continued exploration of vaccine-mediated anti-PC responses, possibly in combination with T-cell targets, in the GPCMV model using MVA vectors. The neutralization of CMV-encoded immunomodulatory gene products is also emerging as a novel future strategy for vaccination [63]. In addition to DISC vaccine studies, subunit vaccine approaches, combining glycoprotein targets and GPCMV immune modulation genes, may merit future consideration in the GPCMV model, and could help inform and direct HCMV vaccine strategies. 

## 5. Conclusions

- A vectored MVA vaccine, constructed by exploiting the 2A peptide system for optimization of multi-subunit herpesvirus vaccines, effectively expresses the GPCMV pentamer subunits.
- Vectored MVA vaccines, expressing the GPCMV homologs of gB, gH/gL and the PC, are immunogenic, and elicit ELISA and neutralizing antibody responses.
- ELISA responses to vaccination were significantly better in the MVA-gpgB vaccine group, compared to the MVA-gp75/gL and MVA-gpPC vaccine groups.
- Neutralization responses were significantly better upon completion of the three-dose vaccine series in the MVA-gpgB vaccine group, compared to the MVA-gpPC vaccine group.
- All vaccine strategies resulted in reduced maternal DNAemia at day 14, following a SG-GPCMV challenge during pregnancy, although reductions were greater for MVA-gpgB vaccine (698-fold), than the MVA-gp75/gL (19.5-fold) and MVA-gpPC (4.9-fold) groups, respectively.
- Preconception vaccination improved pup birth weight and reduced mortality in all groups, but only the gB vaccine resulted in significant reductions in congenital GPCMV transmission.
- Future investigations of glycoprotein vaccines based on MVA vectors are warranted in the GPCMV model.

## Figures and Tables

**Table 1:** National Center for Biotechnology Information (NCBI) Blast of protein sequences with their percent (%) identities and similarities between guinea pig cytomegalovirus (GPCMV) and human cytomegalovirus (HCMV) (taxon ID: 10359). (*) denotes the calculated molecular weight of GP133 and GP133, upon addition of a C-terminal V5-His tag.

GPCMV	HCMV	% Identity	% Similarity	MW (kDa)
GP129	UL128	34	48	22.8
GP131	UL130	29	36	24.3
GP133	UL131	18	29	17.0/19.7 *
GP115	gL	30	49	31.6
GP75	gH	42	64	84.0

**Table 2:** Amino acid sequences of 2A peptide cleavage sites. 2A cleavage is represented by (*); bold sequences are proposed anti-2A epitopes.

Origin	2A	Peptide Sequence
Porcine teschovirus-11	P2A	GSGATNFSLLKQAGDVEENPG*P
*Thosea asigna* virus	T2A	GSGEGRGSLLTCGDVEENPG*P
Foot and Mouth picornavirus	F2A	GSGVKQTLNFDLLKLAGDVESNPG*P
Equine rhinitis A virus	E2A	GSGQCTNYALLKLAGDVESNPG*P

**Table 3:** Mass spectrometry identification of peptides, corresponding to GP133V5-His, GP115 (gL) and GP75 (gH). Protein probability calculations were performed as described in materials and methods. Gel slices correspond to the molecular weight of PC subunits, with each slice receiving a unique letter identifier.

ORF	MW (kDa)	ID	Unique Spectra	Unique Peptide Sequences	% Coverage	Max X_corr_	Probability
GP133_V5-HIS_	19.67	*e*	11	9	43	4.61	100
GP115 (gL)	29.82	*c*	5	4	16	3.70	100
GP75 (gH)	83.85	*a*	10	10	17	4.90	100

**Table 4:** Pup birth outcomes, following maternal pre-conception immunization with MVA expressing GPCMV-antigens.

Vaccine Group	Number Vaccinated	Number Pregnant	Total Pups	Live Pups	Pup Mortality (%)	Pregnancy Duration Post-Challenge (Days)
Venus	8	6	12	3	75	12.0
gH/gL	8	7	24	22	8.3	16.0
PC	8	7	20	19	5.0	11.6
gB	8	7	24	23	4.2	11.8
